# Bacterial persistence induced by salicylate via reactive oxygen species

**DOI:** 10.1038/srep43839

**Published:** 2017-03-10

**Authors:** Tiebin Wang, Imane El Meouche, Mary J. Dunlop

**Affiliations:** 1 School of Engineering, University of Vermont, Burlington, 05405, VT USA

## Abstract

Persisters are phenotypic variants of regular cells that exist in a dormant state with low metabolic activity, allowing them to exhibit high tolerance to antibiotics. Despite increasing recognition of their role in chronic and recalcitrant infections, the mechanisms that induce persister formation are not fully understood. In this study, we find that salicylate can induce persister formation in *Escherichia coli* via generation of reactive oxygen species (ROS). Salicylate-induced ROS cause a decrease in the membrane potential, reduce metabolism and lead to an increase in persistence. These effects can be recovered by culturing cells in the presence of a ROS quencher or in an anaerobic environment. Our findings reveal that salicylate-induced oxidative stress can lead to persistence, suggesting that ROS, and their subsequent impact on membrane potential and metabolism, may play a broad role in persister formation.

Persisters are highly tolerant cells that can survive lethal doses of antibiotics by entering a dormant state^1^. They play an important role in chronic and refractory infections because they can evade antibiotic treatment and re-establish the population after treatment has stopped^2^. In contrast to resistance caused by genetic changes, persisters are genetically identical to antibiotic-susceptible cells. Persisters can be generated by toxin/anti-toxin systems^3^,^4^, the stringent response^5^, and SOS response^6^,^7^. Recent studies have also suggested that there are other more general mechanisms, such as ATP depletion, by which persisters can be formed^8^. Persisters can be generated stochastically^3^,^4^, but mounting evidence suggests that their formation can also be triggered by environmental stresses, such as antibiotics or low nutrient conditions^5^,^6^,^7^. These environmental stimuli affect major cellular processes such as replication, translation, and metabolism, leading to an increase in the fraction of persisters within a bacterial population^5^,^6^,^7^. The mechanisms behind persister formation and the stresses that can cause it are only partially understood.

Salicylate is the active metabolite of aspirin and has been shown to have multiple effects on bacterial physiology^9^. For example, it can inhibit bacterial growth and the expression of genes encoding ATP synthase subunits^10^. Salicylate can also increase the minimum inhibitory concentration of multiple antibiotics through induction of the multiple antibiotic resistance activator MarA^11^. Here we find that salicylate can also induce ROS formation, leading to an increase in bacterial persistence in a MarA-independent fashion.

A few recent studies have suggested that ROS can provide a protective effect against lethal doses of antibiotics by inducing bacterial persistence. However, the mechanisms behind this are not fully understood. For instance, the ROS inducer paraquat can increase antibiotic survival by overexpressing SoxS, and consequently the AcrAB-TolC efflux pumps it controls^12^, though it is not clear whether the pumps are primarily responsible for this effect^13^. In addition, hydrogen peroxide has been used as a direct source of ROS where it offered protection against a lethal dose of ofloxacin^14^. Paradoxically, ROS have also been reported to contribute to the lethality of fluoroquinolones, β-lactams, and aminoglycosides^15^,^16^. The ability of ROS to both protect and kill bacterial cells underscores the complex role that ROS can play in the bacterial response to antibiotics.

In this work, we found that salicylate induces persistence via ROS. Salicylate exposure allows a subpopulation of cells to survive lethal doses of antibiotics even in the absence of MarA. Our findings suggest a pathway by which salicylate induces persistence by increasing ROS, decreasing the membrane potential and metabolism.

## Results

Previous reports have shown that salicylate inhibits bacterial growth^17^, dissipates the proton motive force^18^, and represses genes encoding ATP synthase subunits^10^, therefore, we hypothesized that salicylate might also increase bacterial persistence by decreasing membrane potential and metabolic activity.

**Figure 1:**
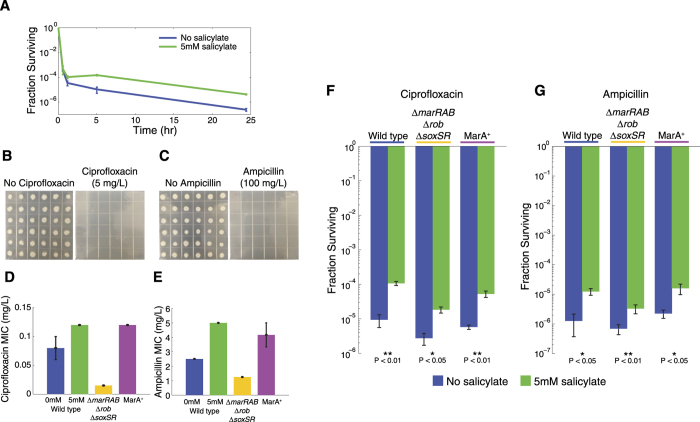
Salicylate increases bacterial persistence. **(A)** Time-dependent killing curves of *E. coli* in the presence of 5 μg/ml ciprofloxacin with or without 5 mM salicylate treatment. Error bars show standard error of n = 3 biological replicates. **(B,C)** Surviving colonies from the 24-hour persister tests with salicylate in (Fig. S2A,B) were spotted on LB only plates or LB supplemented with (**B**) 5 μg/ml ciprofloxacin or (**C**) 100 μg/ml ampicillin. (**D**,**E**) Minimum inhibitory concentration of (**D**) ciprofloxacin and (**E**) ampicillin in wild type strain with and without 5 mM salicylate and in Δ*marRAB* Δ*rob* Δ*soxSR* and MarA^+^ strains. Error bars show standard error from n ≥ 3 biological replicates. **(F,G)** Survival after 6 hours of 5 μg/ml ciprofloxacin or 100 μg/ml ampicillin treatment with and without 5 mM salicylate. Error bars show standard errors from n > 3 biological replicates. We used the Student’s t-test to test for statistical significance. Comparisons are made relative to the untreated condition.

To test our hypothesis, we treated mid-exponential phase cells with salicylate and then exposed them to lethal doses of bactericidal antibiotics. We first used the fluoroquinolone ciprofloxacin, which acts by inhibiting DNA replication^19^ and is effective in all cell growth phases, making it a classical antibiotic for persister isolation^20^. We observed biphasic killing curves indicative of persister formation when treating cultures with a lethal dose of ciprofloxacin (5 μg/ml, 40 × minimum inhibitory concentration) under conditions both with and without 5 mM salicylate treatment (Fig. 1A). In both cases we observed rapid initial killing within the first two hours of incubation with ciprofloxacin, however after this initial period of killing, cells treated with salicylate had a higher survival rate. The difference in the survival rate in the salicylate treated cultures confirmed that salicylate increased persister cell formation. Importantly, this increase in persistence was not due to lower growth of cells treated with salicylate (Fig. S1). We further quantified persister levels by measuring the fraction of cells that survived antibiotic exposure relative to cells prior to exposure. We observed an 11-fold increase in persisters in the salicylate treated cultures after six hours and a 32-fold increase after 24 hours of ciprofloxacin exposure relative to the untreated cultures (Fig. S2A).

In order to investigate the range of salicylate that increases persisters, we treated cells with 1 and 3 mM salicylate before ciprofloxacin exposure. In both cases, we observed an increase, where persister fractions increased with larger salicylate concentrations (Fig. S3).

To test the generality of our findings, we next exposed cells to the β-lactam ampicillin (100 μg/ml, 20 × minimum inhibitory concentration). Ampicillin inhibits cell wall synthesis and is also widely used for persister isolation^3^,^20^. Six hours after treatment with ampicillin, we observed a 10-fold increase in persisters; at 24 hours this ratio increased to 20-fold (Fig. S2B).

To eliminate the possibility that the surviving cells had acquired mutations conferring permanent antibiotic resistance, we plated >100 colonies isolated from our persister assays in conditions with and without ciprofloxacin or ampicillin, and found no colonies that were able to survive antibiotic exposure (Fig. 1B,C). This confirmed that the surviving colonies were not the result of mutations that conferred resistance, but rather were persisters.

Salicylate is the canonical inducer of the *marRAB* operon and induction of MarA’s downstream genes is known to increase the minimum inhibitory concentration for multiple antibiotics. Although salicylate treated cells could tolerate higher doses of antibiotics, the effects were modest and the elevated resistance levels were well below the working concentrations we used in our persister isolation assays (0.12 vs. 5 μg/ml for ciprofloxacin and 5 vs. 100 μg/ml for ampicillin) (Fig. 1D,E). Therefore, we asked whether the increase in persisters was due to MarA and the downstream genes it controls, such as the AcrAB-TolC efflux pumps genes, or if this effect was independent.

To test this we constructed two strains: one where MarA and its homologs Rob and SoxS are knocked out (Δ*marRAB* Δ*rob* Δ*soxSR*) and another where MarA is overexpressed (MarA^+^). In the MarA^+^ strain, the promoter driving the *marRAB* operon has both binding sites for MarR inactivated, mimicking the conditions where salicylate inactivates the MarR repressor, leading to overexpression of MarA. We confirmed that these new strains functioned as expected by measuring the minimum inhibitory concentration of ciprofloxacin and ampicillin. Relative to wild type, the minimum inhibitory concentration decreased in Δ*marRAB* Δ*rob* Δ*soxSR* and increased in MarA^+^ (Fig. 1D,E). The minimum inhibitory concentration of the wild type strain with salicylate treatment was comparable to that of the MarA^+^ strain for both antibiotics, though remained well below the working concentrations we used in our persister isolation assays.

We next measured persister levels under lethal doses of ciprofloxacin and ampicillin and found that salicylate-induced persisters can be generated through a pathway that is independent of MarA (Fig. 1F,G). For Δ*marRAB* Δ*rob* Δ*soxSR*, the basal survival rate decreased compared to the wild type confirming an important role of MarA in antibiotic resistance (Fig. 1F). However, salicylate treatment still significantly increased persister levels compared to the untreated conditions in both Δ*marRAB* Δ*rob* Δ*soxSR* and MarA^+^ strains. After 6 hours of ciprofloxacin exposure we observed a 6.1-fold increase in the Δ*marRAB* Δ*rob* Δ*soxSR* strain and 9.1-fold in the MarA^+^ strain. After 6 hours of ampicillin exposure, we observed a 4.8-fold increase in the Δ*marRAB* Δ*rob* Δ*soxSR* strain and 7.2-fold in the MarA^+^ strain. These results confirm that salicylate can generate persisters via a MarA independent mechanism.

**Figure 2:**
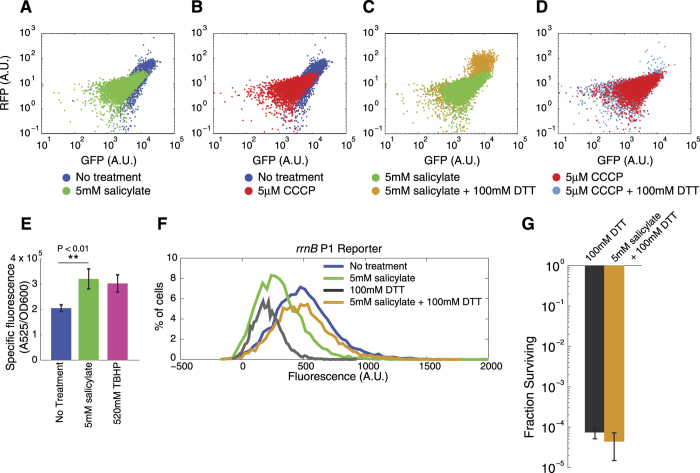
Salicylate induces ROS, dissipates membrane potential, and reduces metabolism. **(A–D)** Red vs. green fluorescence scatter plots showing individual cells treated with the membrane potential indicator DiOC2(3) (**A**) With and without 5 mM salicylate. (**B**) With and without 5 μM CCCP, a protonophore. (**C**) 5 mM salicylate with and without 100 mM DTT, a reducing reagent. (**D**) 5 μM CCCP with and without 100 mM DTT. **(E)** Green fluorescence divided by OD_600nm_ for cultures treated with the general ROS indicator carboxy-H2DCFDA. TBHP is a positive control for ROS. Error bars show standard deviation from n = 3 biological replicates. We used the Student’s t-test to test for statistical significance. **(F)** Fluorescence distributions from the *rrnB* P1 reporter for wild type cells with and without 5 mM salicylate, with 100 mM DTT, and with both 5 mM salicylate and 100 mM DTT. **(G)** Survival after 6 hours of 5 μg/ml ciprofloxacin treatment in the presence of 100 mM DTT with and without 5 mM salicylate. Error bars show standard errors from n = 3 biological replicates.

Salicylate is known to decrease proton motive force in bacteria^18^. Decreasing the proton motive force is an important pathway by which bacterial persisters are formed; previous research has shown that the protonophore CCCP, as well as some toxin/anti-toxin systems can increase persistence by this mechanism^7^,^20^. We reasoned that salicylate-induced persisters could result from a decrease in the membrane potential. To test this, we used the cyanine dye DiOC2(3) which accumulates inside the cell when the membrane potential is intact and shifts its fluorescence emission spectrum when the membrane potential is dissipated. We found that salicylate treatment caused a decrease in the membrane potential relative to untreated cells (Fig. 2A). Results were similar to those in the control group treated with CCCP (Fig. 2B).

We next asked what caused the decrease in membrane potential. To investigate this, we incubated cells with the reducing reagent dithiothreitol (DTT). When we treated cells with both salicylate and DTT, we were able to recover the membrane potential (Fig. 2C). Recovery was not due to the action of DTT alone (Fig. S4). In addition, DTT cannot recover the membrane potential decrease caused by CCCP, which decreases membrane potential directly (not via ROS) by acting as a protonophore (Fig. 2D). This result demonstrates that salicylate-induced ROS play a role in the membrane potential decrease.

We measured ROS directly to further confirm their role by labeling cells with the fluorogenic marker carboxy-H2DCFDA. Cells treated with salicylate showed elevated fluorescence, reaching levels comparable to the positive control incubated with *tert*-Butyl hydroperoxide (TBHP) (Fig. 2E). We also showed an increase in ROS levels after salicylate treatment in the Δ*marRAB* Δ*rob* Δ*soxSR* strain, confirming the independence of this effect from MarA (Fig. S5). These results are in agreement with a previously published finding showing that salicylate can increase ROS generation^21^.

Having established the connection between salicylate, ROS levels, and membrane potential, we next sought to link our findings to bacterial persistence. Because persistence is a state where metabolic activity is decreased, we constructed a reporter for metabolism using the *rrnB* P1 reporter, which measures 16 S rRNA transcriptional level^22^. We observed a decrease in *rrnB* P1 expression in the culture with salicylate, which indicates salicylate can indeed decrease metabolic activity (Fig. 2F). Critically, the decrease in metabolism can be recovered by co-treatment with DTT. DTT alone cannot increase metabolism, and in fact decreased metabolism relative to untreated cells.

The recovery in metabolism due to the combined effect of salicylate and DTT suggests that the increase in persisters in salicylate treated cultures is due to elevated ROS levels. We sought to test this directly by quantifying persister levels in cells treated with DTT alone and DTT with salicylate. Importantly, we found that salicylate could no longer increase persistence in the reducing environment created by DTT (Fig. 2G).

**Figure 3:**
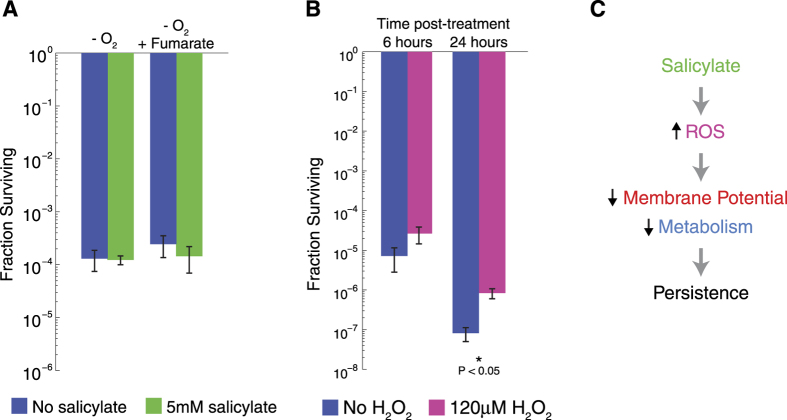
Salicylate-induced persistence requires oxygen. **(A)** Survival with and without 5 mM salicylate after 6 hours of 5 μg/ml ciprofloxacin treatment under anaerobic conditions with and without 40 mM fumarate, an alternative electron acceptor. Error bars show standard errors from n = 3 biological replicates. **(B)** Survival after 6 and 24 hours of 5 μg/ml ciprofloxacin treatment with and without 120 μM H_2_O_2_ as a positive control for oxidative stress. Error bars show standard errors from n = 3 biological replicates. We used the Student’s t-test to test for statistical significance. **(C)** Proposed mechanism for salicylate-induced persistence.

To further confirm our hypothesis that salicylate induces persisters via production of ROS, we measured persister levels using ciprofloxacin in a strict anaerobic environment. Treatment with salicylate no longer increased persister formation in an anaerobic environment (Fig. 3A). This was the case both with and without fumarate, an alternative electron acceptor. Results under anaerobic conditions with ampicillin were similar (Fig. S6). We further confirmed the link between ROS and persister formation by treating cells with hydrogen peroxide (H_2_O_2_) followed by 6 or 24 hours of ciprofloxacin exposure in an aerobic environment (Fig. 3B). We found that treatment with H_2_O_2_ increased persistence in both cases, highlighting the important role of ROS in bacterial persistence.

## Discussion

Here, we showed that salicylate increased bacterial persistence up to 32-fold under lethal doses of bactericidal antibiotics. Although salicylate has a known role in induction of antibiotic resistance through MarA^9^,^23^, the effect is modest and far below the concentrations of antibiotics we used in this study. In addition to the known MarA-dependent resistance, here we demonstrated a MarA-independent role for salicylate in persister cell formation. Bacterial persistence is a more insidious problem than low-level antibiotic resistance, because a small number of persisters can evade antibiotic treatment and go on to recolonize after antibiotic treatment has stopped.

Our results suggest that the incidence of persistence in the presence of salicylate is due to the production of ROS that cause a decrease in membrane potential and metabolism (Fig. 3C). These results are consistent with previous transcriptional studies showing that salicylate can repress translation machinery and ATP synthesis genes^10^. Here, we found that the ROS quencher DTT was able to recover the metabolism levels that were decreased by salicylate. It also recovered the membrane potential and eliminated the effect of salicylate on persister levels. In a parallel set of experiments, we confirmed the role of ROS by growing cells in anaerobic conditions, which eliminated the protective effect of salicylate as well. These findings reveal that salicylate can induce persistence and also present a new pathway by which ROS can increase persister levels via a decrease in membrane potential and metabolism.

There are multiple mechanisms by which ROS may contribute to the formation of persister cells. For example, ROS have been shown to induce efflux pumps via SoxS^12^. Although efflux pump expression can contribute to persister formation^24^, other studies have shown that superoxide can also protect cells from the action of antibiotics even in an efflux pump deficient strain^13^. Moreover, bacterial communication and molecular signaling can lead to increased persistence via oxidative stress response^14^. The role of ROS in antibiotic tolerance and resistance is complex, with recent examples of cases where they are beneficial^12^,^13^,^14^, neutral^25^,^26^, or even detrimental^15^,^16^. Investigations on the effect of ROS in antibiotic resistance, and in particular their role in persistence, remain a rich area for future study. Our results here highlight the importance of ROS in bacterial persistence induced by salicylate and suggest that monitoring ROS levels may provide critical information for understanding persister formation.

## Methods

### Bacterial strains and growth conditions

*E. coli* K-12 MG1655 was cultured in Luria Bertani (LB) medium at 37 °C. The subsequent strains used in this study were all derived from *E. coli* K-12 MG1655.

#### ΔmarRAB Δrob ΔsoxSR strain

We deleted the *marRAB* operon, *rob* gene, and *soxSR* genes using homologous recombination^27^. Primers and further details are provided in Supplementary Methods.

#### MarA^+^ strain

The MarA^+^ strain has the chromosomal copies of the two MarR binding sites in the *marRAB* promoter inactivated, removing negative feedback by MarR. We based the design on transversion mutations from^28^. Further details are provided in Supplementary Methods.

### Persister assays

Cultures were grown aerobically at 37 °C with shaking at 200 rpm overnight and then diluted 1:100 in LB medium. We refer to the time of this dilution as t = 0 hours. The diluted cultures were incubated until t = 3 hours so that cultures reached mid-exponential phase (OD_600nm_ = 0.8 to 1.0). For treated conditions, at t = 2.5 hours we added 5 mM salicylate (Thermo Fisher Scientific S396-500), 120 μM H_2_O_2_ (J. T. Baker #220401), or 100 mM DTT (Thermo Fisher Scientific #AC165680010). At t = 3 hours aliquots from each culture were diluted and plated on LB agar in order to determine the number of colony forming units before antibiotic exposure. We then added 5 μg/ml ciprofloxacin or 100 μg/ml ampicillin and cultures were incubated for either 6 or 24 hours (t = 9 or 27 hours). Cells were then centrifuged and resuspended in sterile phosphate-buffered saline (PBS) to wash out the antibiotics. Washed cultures were diluted and plated on LB agar for 24 hours in order to determine the number of colony forming units after antibiotic exposure. We checked for resistant mutants among the surviving cells by spotting >100 colonies on LB agar and LB agar supplemented with 5 μg/ml ciprofloxacin or 100 μg/ml ampicillin.

### Determining the minimum inhibitory concentration of ampicillin and ciprofloxacin

Overnight cultures of *E. coli* MG1655, Δ*marRAB* Δ*rob* Δ*soxSR*, and MarA^+^ were diluted 1:100 in LB medium. The diluted cultures were incubated at 37 °C with shaking until t = 8 hours. The OD_600nm_ of each culture was then normalized to start new cultures in the presence of antibiotics and 5 mM salicylate, when required. Cells were incubated with increasing concentrations of ciprofloxacin (0 to 0.5 μg/ml in 2-fold dilutions) or ampicillin (0 to 20 μg/ml in 2-fold dilutions) at 37 °C for 24 hours with shaking. The final OD_600nm_ was then determined and cell growth was considered inhibited if OD_600nm_ < 0.1.

### Membrane potential measurements

We used the *Bac*Light™ Bacterial Membrane Potential Kit (Invitrogen #B34950) to measure the membrane potential. Overnight cultures were diluted 1:100 in LB medium and cultured until t = 3 hours. We used the following conditions for the cultures with treatment: 5 mM salicylate was added at t = 1.5 hours, 100 mM DTT was added at t = 2.5 hours, and 5 μM CCCP (R&D Systems #0452500) was added at t = 2.75 hours. The timing of salicylate and DTT treatments were selected to optimize detection while minimizing the impact on cell growth; CCCP timing follows guidelines from the *Bac*Light™ kit. Samples were analyzed using flow cytometry with settings according to the kit instructions to determine the red and green fluorescence values for each sample.

### Reactive oxygen species assay

For the ROS measurements we used the Molecular Probes™ Carboxy-H2DCFDA kit (Invitrogen #C400) according to the manufacturer’s instructions. Overnight cultures were diluted and cultured until t = 3 hours. 5 mM salicylate was added at t = 1 hour to allow for ROS accumulation, and 520 mM TBHP (Acros Organics #180345000) was added at t = 2.25 hours following the protocol developed by Rutheford, *et al*.^29^. Fluorescence was measured at 525 nm using a BioTek Synergy H1m plate reader 10 minutes after adding the dye.

### *rrnB* P1 reporter

We amplified the promoter region of *rrnB* P1^30^ from *E. coli* MG1655 using the forward primer GCCAGGAGCTGAACAATT and the reverse primer TGGTGGCGCATTATAGG. This promoter region was then cloned upstream of *sfgfp* (AddGene #63176) tagged with a *ssrA* degradation tag^31^ on the low-copy (SC101) plasmid pBbS5k^32^ from which the extraneous copy of *lacI* and its promoter have been removed. Kanamycin (30 μg/ml) was added to maintain the resistance marker. The reporter fluorescence level was measured using flow cytometry at t = 3 hours. For the cultures with treatment, we added either 5 mM salicylate and/or 100 mM DTT at t = 2.5 hours. After incubation, cultures were diluted in sterile PBS. Fluorescence levels were measured by flow cytometry using the green and red channels.

### Persister assay in anaerobic conditions

Overnight cultures were diluted 1:100 in LB medium pre-incubated under anaerobic conditions using a gas mixture containing 85% N_2_, 5% CO_2_, and 10% H_2_. Diluted cultures with and without 40 mM fumarate (Acros Organics #AC215531000) were grown in the anaerobic chamber until t = 4 hours at 37 °C to reach mid-exponential phase (OD_600nm_ = 0.8 to 1.0). Salicylate was added at t = 3.5 hours when required. Aliquots from each culture were taken before antibiotic exposure in order to determine the number of colony forming units. 5 μg/ml ciprofloxacin or 100 μg/ml ampicillin was then added to the cultures at t = 4 hours and left until t = 10 hours. Cells were removed from the anaerobic chamber, centrifuged, and resuspended in sterile PBS to wash out the antibiotics. Samples were diluted, plated on LB agar, and incubated aerobically for 24 hours at 37 °C.

## Additional Information

**How to cite this article:** Wang, T. *et al*. Bacterial persistence induced by salicylate via reactive oxygen species. *Sci. Rep.* **7**, 43839; doi: 10.1038/srep43839 (2017).

**Publisher's note:** Springer Nature remains neutral with regard to jurisdictional claims in published maps and institutional affiliations.

## Supplementary Material

Supplementary Information
