# Supplementary material for: Bacterial persistence induced by salicylate via reactive oxygen species
Source: Sci Rep. 2017 Mar 10;7:43839. doi: 10.1038/srep43839 (PMC5345018; doi:10.1038/srep43839)
Supplement: Supplementary Information [file srep43839-s1.pdf]

SUPPLEMENTARY INFORMATION

Bacterial persistence induced by salicylate via reactive oxygen species

Tiebin Wang, Imane El Meouche, Mary J. Dunlop

SUPPLEMENTARY METHODS

Bacterial strains and growth conditions

Δ*marRAB* Δ*rob* Δ*soxSR* strain

We generated a PCR fragment of the kanamycin resistant gene flanked by FRT regions from pKD13 with extensions homologous to the regions adjacent to the operon or gene [1].

For deletion of the *marRAB* operon, we used the forward primer:
CCAGCGATCTGTTCAATGAAATTATTCCATTGGGTCGCTTAATCCATATG
GTGTAGGCTGGAGCTGCTTC
and the reverse primer:
AAGCCCCGAGATGTCGGGGCCAGAACAAACTACATAGCGTGTTGATTAT
AATTCCGGGGATCCGTCGACC.

For deletion of the *rob* gene, we used the forward primer:
CGACGGATCGGAATCAGCAGTTCACAGCGTAGATTAATTGGGCGATCTC
CGTGTAGGCTGGAGCTGCTTC
and the reverse primer:
ATGGATCAGGCCGGCATTATTCGCGACCTTTTAATCTGGCTGGAAGGTC
AATTCCGGGGATCCGTCGACC.

For deletion of the *soxSR* genes, we used the forward primer:
AATTACAGGCGGTGGCGATAATCGCTGGGAGTGCGATCAAACTGCCGAC
GGTGTAGGCTGGAGCTGCTTC
and the reverse primer:
TGTTCATCTTCCAGCAAGCGTGCGCCGGTACCTTCTTCTCCTAAGCGGTC
ATTCCGGGGATCCGTCGACC.

Bold letters indicate the homologous recombination extensions. We then followed the protocol from [1] using the phage lambda recombinase harbored on the low copy plasmid pSIM6. After recombination, the resistance marker gene was removed using the pCP20 helper plasmid encoding the FLP recombinase.

MarA$^+$ strain

The transversion mutations were derived from [2], where they are listed as "TV -14 to -18" and "TV +11 to 15." To construct this strain we synthesized a DNA fragment containing a modified version of the *marRAB* promoter:
GCATCGCATTGAACAAAACTTGAACCGATTTAGCAAAACGTGGCATCGGTCA
ATTCATTCATTTGACTTATACTTGCCTG**TTACC**TATTATCCCCTGCAACTAATT
AC**GGTAA**AGGGCAACTAATGTGAAAAGTACCAGCGATCTGTTCAATGAAATT
ATT.
Underlined letters indicate the MarR binding sites and bold letters indicate the transversion mutations. We introduced this modified promoter via homologous recombination following [1], then cured the resistance marker. To accomplish this, we ligated the modified *marRAB* promoter given above to the kanamycin resistance gene

flanked by FRT regions from pKD13. The ligated fragment containing the kanamycin resistance gene followed by the modified *marRAB* promoter was then amplified using the forward primer:

GGGGTAAACAAGGATAAAGTGTCACTCTTTAGCTAGCCTTGCATCGCAT TGTGTAGGCTGGAGCTGCTTC

and the reverse primer:

AATAATTTCATTGAACAGATCGCTGGT.

Bold letters indicate the homologous recombination extensions. We followed the protocol for recombination and curing of the kanamycin marker as described above.

SUPPLEMENTARY FIGURES

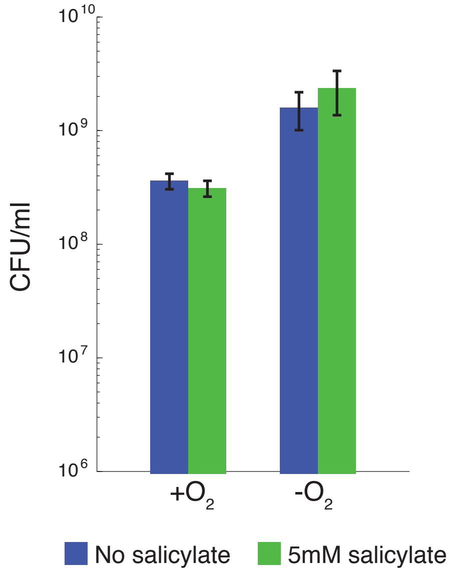

Figure S1: Salicylate exposure does not affect initial cell counts before addition of antibiotics.

Colony forming units per milliliter (CFU/ml) for cultures with and without 5 mM salicylate under aerobic conditions and anaerobic conditions before addition of antibiotics ($t = 3$ hours in aerobic conditions and $t = 4$ hours in anaerobic conditions). Error bars show standard errors from $n = 12$ biological replicates under aerobic conditions and from $n = 6$ biological replicates under anaerobic conditions.

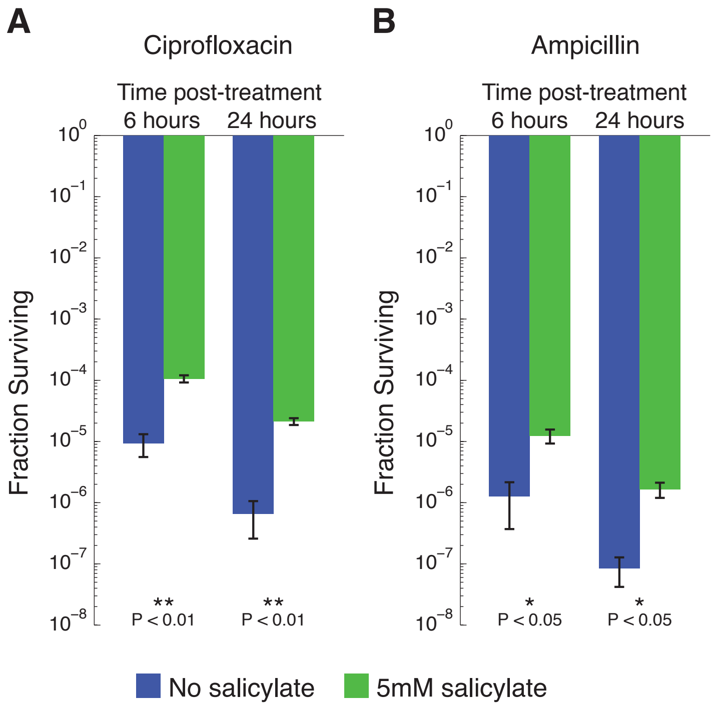

Figure S2: Salicylate increases survival rate after 6 and 24 hours of ciprofloxacin or ampicillin treatment.

Fraction of E. coli MG1655 surviving after 6 and 24 hours of (A) 5 µg/ml ciprofloxacin or (B) 100 µg/ml ampicillin treatment with and without 5 mM salicylate. Error bars show standard error of n = 3 biological replicates. We used the Student's t-test to test for statistical significance.

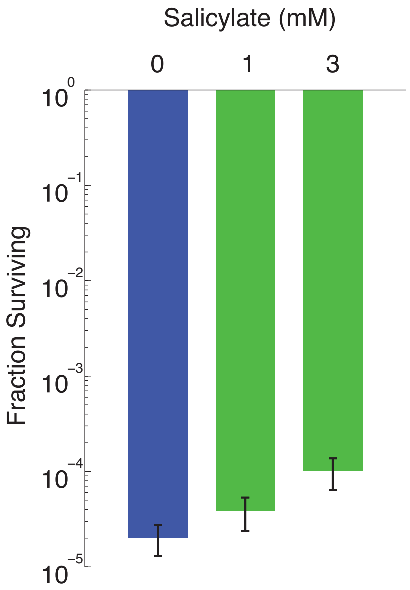

Figure S3: 1 and 3 mM salicylate increase persister levels.
Survival after 6 hours of 5 μg/ml ciprofloxacin treatment with and without 1 or 3mM salicylate. Error bars show standard errors from n = 3 biological replicates.

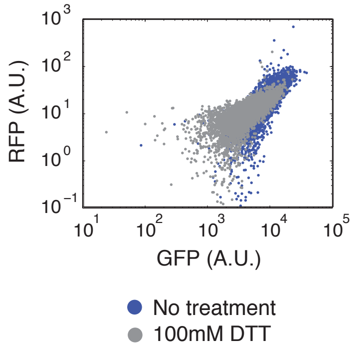

Figure S4: Effect of DTT on membrane potential.
Red vs. green fluorescence scatter plots showing individual cells treated with the membrane potential indicator DiOC2(3) with and without 100 mM DTT.

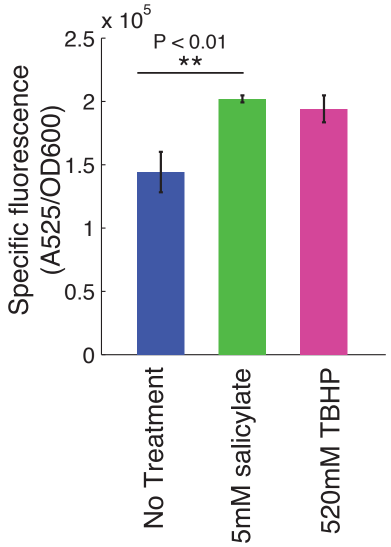

Figure S5: Salicylate induces ROS in Δ*marRAB* Δ*rob* Δ*soxSR* strain.
Green fluorescence divided by OD_{600nm} for Δ*marRAB* Δ*rob* Δ*soxSR* cultures treated with the general ROS indicator carboxy-H2DCFDA. TBHP is a positive control for ROS. Error bars show standard deviation from n = 3 biological replicates. We used the Student's t-test to test for statistical significance.

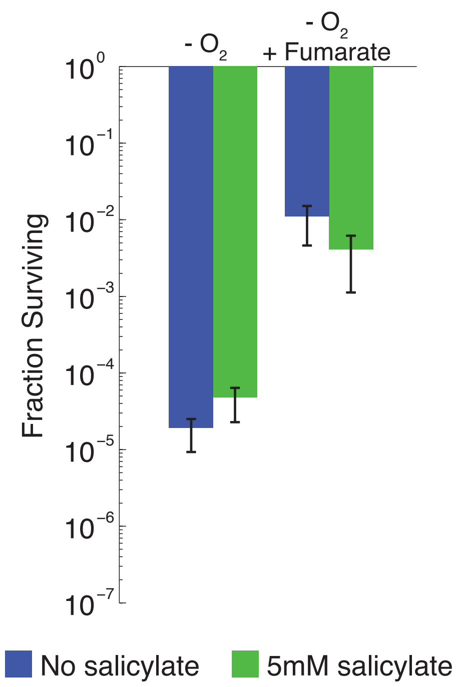

Figure S6: Salicylate-induced persistence in ampicillin requires oxygen.
Survival with and without 5 mM salicylate after 6 hours of 100 µg/ml ampicillin treatment under anaerobic conditions with and without 40 mM fumarate. Error bars show standard errors from n = 3 biological replicates.

SUPPLEMENTARY REFERENCES

1 Datsenko, K. A. & Wanner, B. L. One-step inactivation of chromosomal genes in *Escherichia coli* K-12 using PCR products. *Proceedings of the National Academy of Sciences of the United States of America* **97**, 6640-6645, doi:10.1073/pnas.120163297 (2000).

2 Martin, R. G. & Rosner, J. L. Transcriptional and translational regulation of the *marRAB* multiple antibiotic resistance operon in *Escherichia coli*. *Molecular microbiology* **53**, 183-191, doi:10.1111/j.1365-2958.2004.04080.x (2004).
